# Building a Prediction Model for Radiographically Confirmed Pneumonia in Peruvian Children: From Symptoms to Imaging

**DOI:** 10.1016/j.chest.2018.09.006

**Published:** 2018-10-03

**Authors:** Farhan Pervaiz, Miguel A. Chavez, Laura E. Ellington, Matthew Grigsby, Robert H. Gilman, Catherine H. Miele, Dante Figueroa-Quintanilla, Patricia Compen-Chang, Julio Marin-Concha, Eric D. McCollum, William Checkley

**Affiliations:** a Division of Pulmonary and Critical Care, School of Medicine, Johns Hopkins University, Baltimore, MD; b Biomedical Research Unit, A.B. PRISMA, Lima, Peru; c Department of Pulmonary and Sleep Medicine, Seattle Children’s Hospital, University of Washington, Seattle, WA; d Program in Global Disease Epidemiology and Control, Department of International Health, Bloomberg School of Public Health, Johns Hopkins University, Baltimore, MD; e Instituto Nacional de Salud del Nino, Lima, Peru; f Department of Pediatrics, Eudowood Division of Pediatric Respiratory Sciences, School of Medicine Johns Hopkins University, Baltimore, MD

**Keywords:** auscultation, lung ultrasound, oxyhemoglobin saturation, pneumonia, prediction models, AUC, area under the curve, CXR, chest radiography, LUS, lung ultrasound, Spo_2_, oxyhemoglobin saturation, WHP, World Health Organization

## Abstract

**Background:**

Community-acquired pneumonia remains the leading cause of death in children worldwide, and current diagnostic guidelines in resource-poor settings are neither sensitive nor specific. We sought to determine the ability to correctly diagnose radiographically confirmed clinical pneumonia when diagnostics tools were added to clinical signs and symptoms in a cohort of children with acute respiratory illnesses in Peru.

**Methods:**

Children < 5 years of age with an acute respiratory illness presenting to a tertiary hospital in Lima, Peru, were enrolled. The ability to predict radiographically confirmed clinical pneumonia was assessed using logistic regression under four additive scenarios: clinical signs and symptoms only, addition of lung auscultation, addition of oxyhemoglobin saturation (Spo_2_), and addition of lung ultrasound.

**Results:**

Of 832 children (mean age, 21.3 months; 59% boys), 453 (54.6%) had clinical pneumonia and 221 (26.6%) were radiographically confirmed. Children with radiographically confirmed clinical pneumonia had lower average Spo_2_ than those without (95.9% vs 96.6%, respectively; *P* < .01). The ability to correctly identify radiographically confirmed clinical pneumonia using clinical signs and symptoms was limited (area under the curve [AUC] = 0.62; 95% CI, 0.58-0.67) with a sensitivity of 66% (95% CI, 59%-73%) and specificity of 53% (95% CI, 49%-57%). The addition of lung auscultation improved classification (AUC = 0.73; 95% CI, 0.69-0.77) with a sensitivity of 75% (95% CI, 69%-81%) and specificity of 53% (95% CI, 49%-57%) for the presence of crackles. In contrast, the addition of Spo_2_ did not improve classification (AUC = 0.73; 95% CI, 0.69-0.77) with a sensitivity of 40% (95% CI, 33%-47%) and specificity of 72% (95% CI, 68%-75%) for an Spo_2_ ≤ 92%. Adding consolidation on lung ultrasound was associated with the largest improvement in classification (AUC = 0.85; 95% CI, 0.82-0.89) with a sensitivity of 55% (95% CI, 48%-63%) and specificity of 95% (95% CI, 93%-97%).

**Conclusions:**

The addition of lung ultrasound and auscultation to clinical signs and symptoms improved the ability to correctly classify radiographically confirmed clinical pneumonia. Implementation of auscultation- and ultrasound-based diagnostic tools can be considered to improve diagnostic yield of pneumonia in resource-poor settings.

Pneumonia remains the most common infectious cause of morbidity and mortality in young children worldwide.^1^ Most of the 1.1 million deaths in children < 2 years of age occur in resource-limited settings.^2^ Early and accurate diagnosis of bacterial pneumonia presents a major challenge toward successful treatment. Current international guidelines rely on clinical presentation and physical examination, with imaging used in ambiguous or severe cases.^3^, ^4^ A lack of trained physicians and access to diagnostic tools, such as laboratory tests and imaging, make it difficult to follow international guidelines in resource-limited settings.

The World Health Organization (WHO) developed a pneumonia case management algorithm for resource-limited settings, allowing diagnosis based on symptoms and clinical signs.^5^ This algorithm has been shown to have low diagnostic specificity.^6^, ^7^, ^8^ Furthermore, no individual clinical features, including those in the WHO case management algorithm, were sufficient to reliably predict radiographically confirmed pneumonia.^9^ Although chest radiography (CXR) is a standard diagnostic tool for the identification of pneumonia, it has poor validity.^10^ Without a standardized training approach, such as the WHO CXR methodology, CXR also has high interobserver variability, and current clinical guidelines do not require a CXR for the diagnosis of pneumonia.^4^ There is evidence that lung auscultation and pulse oximetry improve the ability to correctly identify pneumonia^11^, ^12^; however, a recent prospective study had mixed results with pulse oximetry improving diagnosis but not auscultation.^13^ Lung ultrasound (LUS) has been shown to have good sensitivity and specificity compared with CXR.^14^, ^15^, ^16^, ^17^

Previous studies have attempted to develop predictive models in children with suspicion of pneumonia.^18^, ^19^, ^20^, ^21^, ^22^ Current algorithms have not been reliable and have been limited by small samples or the exclusion of common pediatric respiratory diseases such asthma or bronchiolitis.^21^, ^22^ We sought to assess the diagnostic value of clinical prediction models based on lung auscultation, pulse oximetry, and LUS to identify radiographically confirmed clinical pneumonia in Peruvian children < 5 years of age. This assessment may elucidate the value of implementing these clinical tools where CXR may not be available or appropriate in the diagnosis of pneumonia.

## Methods

### Study Design

We consecutively enrolled children aged 2 to 59 months presenting to the ED, inpatient wards, and outpatient clinics with an acute respiratory illness at the Instituto Nacional de Salud del Niño in Lima, Peru, between January 2012 and September 2013.^23^ The Instituto Nacional de Salud del Niño is the largest freestanding pediatric hospital in Lima. It is a public government-run hospital (www.insn.gob.pe) serving predominantly low-income populations, and it is also a national referral center. We excluded children with a history of significant heart disease or chronic respiratory disease other than asthma and children who required invasive airway management. We also recruited 230 children without any acute illness,^23^ but we limited the use of their data to oxyhemoglobin saturation (Spo_2_) values in this analysis. This cohort of children was used in a previous LUS validation study,^16^ and study protocol was published elsewhere.^23^ The study was approved by the institutional review board committees of the Instituto Nacional de Salud del Niño (Lima, Peru) (No. CL-4311), A.B. PRISMA (Lima, Peru) (No. CE1457.11), and the Johns Hopkins School of Medicine (Baltimore, MD) (No. 64148). Written informed consent was obtained from a parent or guardian prior to enrollment into the study.

### Data Collection

Child participants who met inclusion criteria underwent a standard clinical assessment, after consent was obtained from their parents, for signs and symptoms, including lung auscultation, pulse oximetry, and imaging. Clinical assessment, including auscultation, was conducted by the treating pediatrician. A study team member recorded the clinical findings, including auscultation findings, vital signs, presenting history, and Spo_2_.^23^ All children underwent LUS and had an anterior-posterior CXR taken. Spo_2_ was assessed using pediatric probes on either Rad 5v pulse oximeters (Masimo Corp) or, in few instances, the peripheral pulse oximeters available at Instituto Nacional de Salud del Niño. Lung auscultation was performed on the anterior and posterior zones of the thorax, with the patient supine or upright, as previously described.^23^ Pediatricians conducting auscultation were asked to report the presence of the following findings: crackles, wheeze, decreased breath sounds, or bronchial breath sounds. Ausculatory findings were obtained with acoustic stethoscopes.

### Definitions

We used WHO growth standards to define wasting (weight-for-height *z* score < −2 SD), stunting (height-for-age *z* score < −2 SD), and severe malnutrition (weight-for-height *z* score of ≤ −3 SD).^24^ We used age-specific respiratory rate cutoffs to define tachypnea: ≥ 50 breaths/min for children 2 to 11 months of age and ≥ 40 breaths/min for children 12 to 59 months of age.^4^ Tachycardia was defined as ≥ 190 beats/min for children 2 to 11 months of age and ≥ 140 beats/min for children 12 to 59 months of age.^4^ Pulse oximetry was included as a continuous variable, but we conducted sensitivity analyses with Spo_2_ cutoffs of ≤ 92% and ≤ 95%.

### Pneumonia

The definitions of clinical pneumonia, asthma, bronchiolitis, or an upper respiratory tract infection were based on standard of care, using patient history and physical examination, including Spo_2_ and CXR results. A clinical diagnosis was made by the treating pediatrician. Children had WHO-defined pneumonia if they had an acute presentation of either cough or difficulty breathing and also had either lower chest wall indrawing or age-specific tachypnea.^4^ Severe pneumonia or very severe disease was defined as WHO pneumonia with at least one of the following danger signs: persistent vomiting, convulsions, lethargy, no oral intake, stridor, or severe malnutrition.^25^ Severe clinical pneumonia was defined as a clinical diagnosis of pneumonia, by the treating pediatrician, and the presence of at least one of the danger signs previously listed.^25^

### LUS

Study children received a complete LUS using a MicroMaxx portable ultrasound machine (Sonosite/FujiFilm) with an HFL38/13-6 MHz linear transducer. LUS assessment was conducted by one of three trained general practitioners following a standardized protocol developed using international recommendations.^16^, ^26^ Interpretation and conduct of LUS were performed independent of clinical evaluation and CXR findings.^16^ We defined pneumonia on LUS as the presence of a hypoechoic area consistent with a consolidation and occupying of more than one intercostal space in longitudinal view, or a smaller consolidation with a pleural effusion, and interstitial abnormalities was defined as three or more B lines within a single acoustic window. We required agreement by two of three ultrasound readers for a final LUS diagnosis.^16^

### Radiographic Pneumonia

We obtained anteroposterior CXR on all children with an acute respiratory illness. Radiographic pneumonia was defined as the presence of a lobar consolidation with or without pleural effusion.^27^ All chest radiographs were reviewed by two members of a team of three expert pediatric radiologists blinded to clinical information and results from LUS.^16^ Radiographic diagnosis was made as a consensus of the team using a standardized protocol, as previously described.^16^

### Biostatistical Methods

Our primary objective was to assess the ability of different diagnostic algorithms to correctly classify children diagnosed with clinical pneumonia that is corroborated by the finding of a lobar consolidation on CXR. As such, we compared each additive clinical scenario against radiographically confirmed clinical pneumonia. We evaluated the following four additive scenarios: WHO-defined pneumonia,^5^ addition of lung auscultation findings, addition of Spo_2_ by pulse oximetry, followed by the addition of LUS findings. We used multivariable logistic regression to model the presence of radiographically confirmed clinical pneumonia as a function of the four additive scenarios, adjusted for malnutrition (both wasting and stunting), having tachycardia, and a having previous history of pneumonia.

We used logistic regression to calculate a concordance statistic (C statistic), which is statistically equivalent to the area under the curve (AUC).^28^ Models with a higher AUC did better at identifying radiographically confirmed clinical pneumonia. Analyses were performed using STATA version 13 (Stata Corp) and R (The R Foundation).

## Results

### Participant Characteristics

There were 832 children recruited to the study and who underwent diagnostic imaging for pneumonia. Two children were missing clinical data (< 1%) and were excluded from the analysis. We summarized participant characteristics in Table 1. Mean participant age was 21.3 months, 59% of which were boys, 8% were wasted, and 17% were stunted. The study primarily included an inner city population that is low to middle income. We summarized socioeconomic status in Table 1. Overall, final clinical diagnoses, as reported by the treating pediatrician, were as follows: 453 (55%) had clinical pneumonia, 133 (16%) had asthma, 103 (12%) had bronchiolitis, and 143 (17%) had an upper respiratory infection. Radiologists identified 221 consolidations (27%) and 264 interstitial opacities (32%) on chest radiographs in children. A total of 191 children (23%) met criteria for radiographically confirmed clinical pneumonia and 429 children (51.6%) met criteria for WHO-defined pneumonia.

**Table 1:** Demographic Information and Clinical Characteristics According to Study Group

Characteristics	Full Sample	2-11 mo of Age	12-59 mo of Age
Demographic characteristics			
Sample size	832 (100)	39 (322)	61 (510)
Age, mo	21.3 ± 16.2	6.5 ± 2.8	30.6 ± 14.1
No. of boys	59 (488)	60 (193)	58 (295)
Social demographics			
No. of people in household	5.0 ± 0.07	5.3 ± 0.11	4.9 ± 0.09
No. of people in household			
< 3	3 (22)	2 (5)	3 (17)
3-6	78 (648)	76 (243)	80 (405)
7-10	17 (138)	20 (64)	15 (74)
> 10	3 (22)	3 (9)	3 (13)
Employment status of parents			
Both parents employed	23 (193)	13 (43)	30 (150)
Only father employed	72 (596)	82 (260)	66 (336)
Only mother employed	2 (17)	2 (7)	2 (10)
Neither parent employed	2 (20)	3 (9)	2 (11)
Father education level, y			
< 6	2 (17)	2 (7)	2 (10)
6-10	15 (120)	18 (56)	13 (64)
11-12	59 (483)	62 (195)	57 (288)
> 12	22 (182)	16 (51)	26 (131)
Mother education level, y			
< 6	3 (25)	4 (14)	2 (11)
6-10	25 (211)	28 (91)	24 (120)
11-12	50 (413)	53 (171)	48 (242)
> 12	22 (179)	14 (45)	26 (134)
Water supply			
Home water supply	91 (755)	88 (283)	93 (472)
External water supply	9 (77)	12 (39)	7 (38)
Toilet waste elimination			
Connection to city drainage	90 (746)	86 (277)	92 (469)
Home septic tank	< 1 (1)	0 (0)	< 1 (1)
Latrine	10 (85)	14 (45)	8 (40)
Clinical characteristics			
Weight-for-height *z* score	0.26 ± 1.70	0.48 ± 1.68	0.12 ± 1.69
% < −2 SD	8 (69)	7 (21)	9 (48)
Height-for-age *z* score	−0.42 ± 1.95	−0.48 ± 1.96	−0.39 ± 1.95
% < −2 SD	17 (140)	19 (61)	15 (79)
Symptoms			
Cough	99 (825)	99 (321)	99 (504)
Difficulty breathing	84 (696)	88 (282)	81 (414)
Fever	64 (529)	64 (205)	64 (324)
Chest indrawing	36 (300)	47 (150)	29 (150)
Temperature (°C)	36.8 ± 0.65	36.8 ± 0.60	36.8 ± 0.68
No. with ≥ 38.0°C	9 (74)	8 (26)	9 (48)
Heart rate	130 ± 18	135 ± 17	126 ± 18
Tachycardia	15 (126)	0 (0)	25 (126)
Respiratory rate	39 ± 12	44 ± 12	36 ± 11
Tachypnea	36 (301)	31 (99)	40 (202)
Oxygen saturation	96 ± 3	97 ± 3	96 ± 3
No. ≤ 95%	31 (259)	31 (99)	31 (160)
No. ≤ 92%	9 (73)	8 (25)	9 (48)
Auscultation findings			
Wheeze	45 (373)	46 (148)	44 (225)
Crackles	53 (445)	57 (183)	51 (262)
Decreased breath sounds	12 (98)	8 (27)	14 (71)

Values are mean ± SD or %. (No).

### Distribution of Spo_2_ by Pneumonia Status

**Figure 1:**
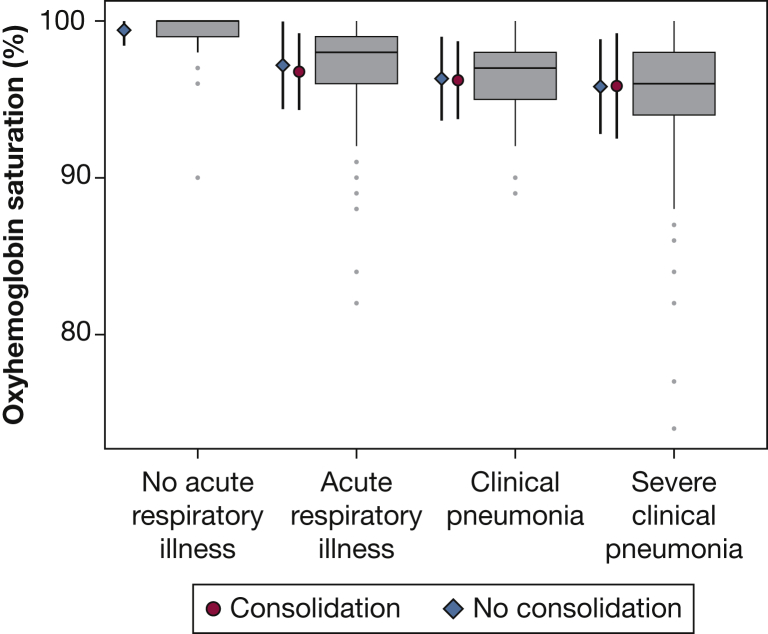
Oxyhemoglobin saturation (Spo_2_) among children by acute respiratory illness status. Boxplots, from left to right, represent Spo_2_ values for children without an acute respiratory illness, with an acute respiratory illness that was not pneumonia (asthma, bronchiolitis, or upper respiratory infections), with clinical pneumonia, and with severe clinical pneumonia. The gray dots represent outliers (ie, values that lie > 1½ times the interquartile range). Diamonds/circles and vertical bars to the left of each boxplot represent the mean Spo_2_ and corresponding 95% CI, respectively, stratified by chest radiography (CXR) findings (diamonds show CXR without a consolidation, circles show CXR with a consolidation).

We plotted the distribution of Spo_2_ values by categories of acute respiratory illness, ranging from none to having severe pneumonia, and stratified by CXR findings (Fig 1). Mean Spo_2_ was lowest in children with a clinical diagnosis of pneumonia, followed by children with either asthma, bronchiolitis, or an upper respiratory tract infection. It was highest among children without an acute illness. Overall, mean Spo_2_ was lower in children with clinical pneumonia than in those who did not have clinical pneumonia (95.9% vs 97.1%, respectively; *P* < .001). There was no difference in Spo_2_ values between children with nonsevere clinical pneumonia and those with severe clinical pneumonia (95.9% vs 95.5%, respectively; *P* = .78). However, a difference was seen between children with WHO-defined pneumonia and those with WHO-defined severe pneumonia (96.1% vs 95.3%, respectively; *P* = .02). Additionally, no difference was found between children with radiographically confirmed clinical pneumonia and those with clinical pneumonia without a consolidation (95.9% vs 95.9%, respectively; *P* = .96).

### Classification of Radiographically Confirmed Pneumonia

**Figure 2:**
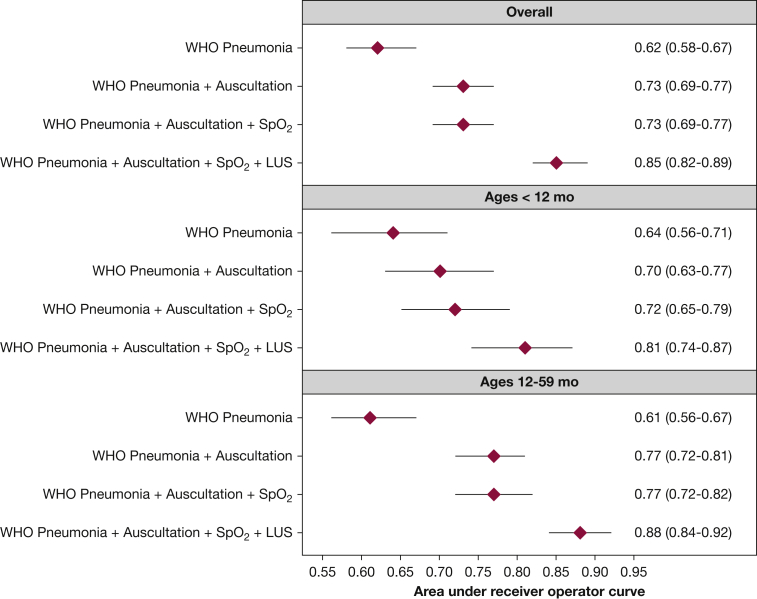
Area under the curve (AUC) (C statistic) for each of the four additive clinical scenarios used to classify radiographically confirmed clinical pneumonia, stratified by age group. We plotted AUCs and corresponding 95% CIs derived from multivariable logistic regression models for each additive clinical scenario (described in y axis), for all children in the sample (first panel) and stratified by age group: 2 to 11 mo of age (middle panel) and 12 to 59 mo of age (bottom panel). We also present numerical AUCs and 95% CIs for each row. LUS = lung ultrasound; Spo_2_ = oxyhemoglobin saturation; WHO = World Health Organization.

**Figure 3:**
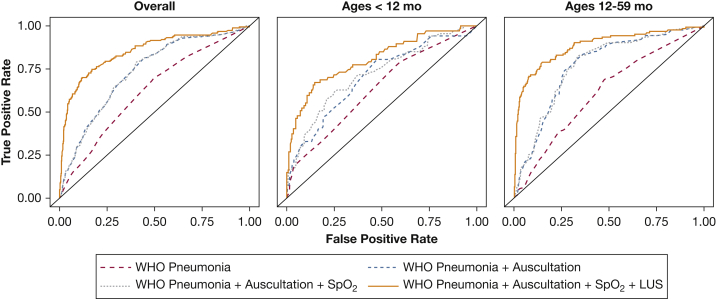
Receiver operating characteristic (ROC) curves for each of the four additive clinical scenarios used to classify radiographically confirmed clinical pneumonia, stratified by age group. We plotted ROC curves derived from multivariable logistic regression models for each additive clinical scenario (described in y axis), for all children in the sample (left panel) and stratified by age group: 2 to 11 mo of age (middle panel) and 12 to 59 mo of age (right panel). See Figure 2 legend for expansion of other abbreviations.

**Figure 4:**
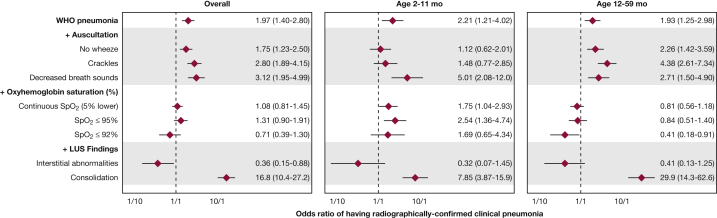
Forest plot of the odds of having radiographically confirmed clinical pneumonia using four types of clinical tools. We plotted the adjusted OR of having radiographically confirmed clinical pneumonia for each four clinical tools in an additive scenario using overall study sample, and then stratified by age groups (2-11 and 12-59 mo of age). Adjusted ORs are represented with diamonds and 95% CIs are represented by horizontal lines. We show the additive scenarios on the y axis. Four sets of logistic regression models were built. The first model included a composite for variables to WHO pneumonia, and adjusted for confounders (medical history of pneumonia, age-specific tachycardia, and weight-for-height and height-for-age z scores). The second model included WHO pneumonia, three auscultatory variables (absence of wheezes, presence of crackles, and decreased breath sounds), and vide supra confounders. The third model was a set of models that included WHO pneumonia, the three auscultatory variables, Spo_2_ expressed three different ways, and vide supra confounders. Specifically, we ran three independent models with Spo_2_ as a continuous variable, and Spo_2_ with the thresholds of ≤ 95% and ≤ 92%. The fourth model included WHO pneumonia, the three auscultatory variables, continuous Spo_2_, two lung ultrasound variables (interstitial abnormalities or consolidation), and vide supra confounders. Adjusted ORs and 95% CIs are also presented numerically for each row. See Figure 2 legend for expansion of abbreviations.

We summarized the diagnostic validity for each individual clinical tool (Table 2) and plotted AUCs (Fig 2) and corresponding receiver operating characteristic curves (Fig 3) for the different diagnostic tools based on the described four additive scenarios to classify pneumonia when using radiographically confirmed clinical pneumonia. WHO-defined pneumonia had a 66% sensitivity and 53% specificity for correctly identifying radiographically confirmed clinical pneumonia. The presence of cough or shortness of breath with intercostal indrawing or age-specific tachypnea, crackles on auscultation, and decreased breath sounds on auscultation had sensitivities > 60%, whereas decreased breath sounds on auscultation, Spo_2_ ≤ 92%, and consolidation on LUS had specificities > 90% (Table 2). Consolidation on LUS had the highest positive likelihood ratio for radiographically confirmed clinical pneumonia, whereas consolidation on LUS and presence of crackles had the lowest negative likelihood ratio. As noted in Figure 2, the use of WHO-defined pneumonia was limited in its ability to classify radiographically confirmed clinical pneumonia. The addition of lung auscultation improved the classification of radiographically confirmed clinical pneumonia, with decreased breath sounds, presence of crackles, and absence of wheezes independently associated (Fig 4). The addition of pulse oximetry to identify hypoxemia as a continuous variable (Fig 2) did not improve the classification of radiographically confirmed clinical pneumonia beyond WHO-defined pneumonia and lung auscultation. Additionally, no improvement in classification is seen using hypoxemia cutoffs of Spo_2_ ≤ 92% (AUC = 0.73; 95% CI, 0.69-0.77) or ≤ 95% (AUC = 0.73; 95% CI, 0.69-0.77). Both hypoxemia cutoffs, ≤ 92% and ≤ 95%, were not associated with radiographically confirmed clinical pneumonia (Fig 4). Finally, the addition of Spo_2_ alone to WHO-defined pneumonia did not improve classification (AUC = 0.63; 95% CI, 0.59-0.67).

**Table 2:** Assessment of Diagnostic Validity of Each Clinical Tool

Diagnostic Tools	Se % (95% CI)	Sp % (95% CI)	LR+ (95% CI)	LR– (95% CI)	PPV % (95% CI)	NPV % (95% CI)
WHO pneumonia	66.3 (59.1-73.0)	52.7 (48.7-56.6)	1.40 (1.23-1.60)	0.64 (0.52-0.79)	29.4 (25.1-33.9)	84.0 (80.1-87.5)
Auscultation findings						
Presence of crackles	75.3 (68.5-81.2)	53.0 (49.0-56.9)	1.60 (1.43-1.80)	0.47 (0.36-0.60)	32.2 (27.9-36.8)	87.8 (84.1-90.9)
Absence of wheezes	64.2 (57.0-71.0)	47.3 (43.4-51.3)	1.22 (1.07-1.39)	0.76 (0.61-0.93)	26.6 (22.6-30.9)	81.7 (77.4-85.5)
Decreased breath sounds	23.2 (17.4-29.8)	91.6 (89.1-93.6)	2.74 (1.91-3.95)	0.84 (0.77-0.91)	44.9 (34.8-55.3)	80.1 (77.0-82.9)
Oxyhemoglobin saturation ≤ 95%	9.5 (5.7-14.6)	91.4 (89.0-93.5)	1.10 (0.66-1.83)	0.99 (0.94-1.04)	24.7 (15.3-36.1)	77.3 (74.1-80.2)
Oxyhemoglobin saturation ≤ 92%	40.0 (33.0-47.3)	71.6 (67.9-75.0)	1.41 (1.14-1.74)	0.84 (0.74-0.95)	29.5 (24.0-35.4)	80.1 (76.6-83.3)
Consolidation on lung ultrasound	55.3 (47.9-62.5)	95.0 (93.0-96.6)	11.05 (7.70-15.9)	0.47 (0.40-0.55)	76.6 (68.7-83.4)	87.7 (85.1-90.1)

LR+ = positive likelihood ratio, LR– = negative likelihood ratio; NPV = negative predictive value; PPV = positive predictive value; Se = sensitivity; Sp = specificity; WHO = World Health Organization.

LUS contributed to the largest improvement in the classification of radiographically confirmed clinical pneumonia. When LUS alone, without auscultation and Spo_2_, was added to WHO-defined pneumonia, it improved the classification of radiographically confirmed clinical pneumonia (AUC = 0.82; 95% CI, 0.78-0.85). Consolidation on LUS was associated with radiographically confirmed clinical pneumonia (Fig 4). Finding interstitial abnormalities on LUS was indicative of not having radiographically confirmed clinical pneumonia. The addition of Spo_2_ and LUS, without lung auscultation, improved classification of radiographically confirmed pneumonia beyond clinical signs and symptoms (AUC = 0.82; 95% CI, 0.79-0.86). However, the model that included lung auscultation, with Spo_2_ and LUS, had better discrimination (AUC = 0.85 vs AUC = 0.82).

### Subgroup Analyses

In children 12 to 59 months of age, the use of WHO-defined pneumonia resulted in poor classification of radiographically confirmed clinical pneumonia (Fig 2). The addition of lung auscultation improved classification (Fig 2), with crackles, decreased breath sounds, and absence of wheezes independently associated with radiographically confirmed clinical pneumonia (Fig 4). The addition of pulse oximetry to identify hypoxemia did not improve classification (Fig 2) and was not independently associated with radiographically confirmed clinical pneumonia (Fig 4). LUS contributed the largest improvement in the classification of radiographically confirmed clinical pneumonia.

In infants 2 to 11 months of age, the use of WHO-defined pneumonia also resulted in poor classification (Fig 2). The addition of lung auscultation improved classification, albeit less so than in children 12 to 59 months of age (Fig 2). When the contributions of lung auscultation were also assessed independently, we found that the presence of crackles and absence of wheezes were not associated with radiographically confirmed clinical pneumonia, whereas decreased breath sounds were associated with radiographically confirmed clinical pneumonia (Fig 4). The addition of pulse oximetry resulted in an improvement in the classification of radiographically confirmed clinical pneumonia (Fig 2). A lower Spo_2_ was associated with a higher odds of having radiographically confirmed clinical pneumonia (Fig 4). Finally, LUS again was associated with the largest improvement in the classification of radiographically confirmed clinical pneumonia (Fig 2).

## Discussion

We found that the WHO definition of pneumonia based on clinical symptoms and signs alone had poor discrimination for radiographically confirmed clinical pneumonia among Peruvian children who presented with an acute respiratory illness. Although children with pneumonia had lower Spo_2_, the use of pulse oximetry to identify hypoxemia did not add value, above WHO-defined pneumonia, to the classification of radiographically confirmed clinical pneumonia in the overall population. In contrast, both the use of auscultation or LUS improved the classification of children with radiographically confirmed clinical pneumonia.

Lung auscultation remains an important component of pneumonia diagnosis with more predictive accuracy than an initial clinical assessment alone. In the study population, the presence of crackles, decreased breath sounds, and absence of wheezes were all important predictors for radiographically confirmed clinical pneumonia. This is consistent with other studies where auscultation is a useful predictor of radiographically confirmed pneumonia.^29^ Presence of physicians, trained personnel, or even a device^30^ that identifies lung sounds may be critical for increasing the accuracy of a clinical algorithm for the diagnosis of pneumonia in resource-limited settings.

The use of pulse oximetry to identify pneumonia is supported by studies showing that a low Spo_2_ may help to identify more cases of pneumonia than a clinical approach alone.^12^, ^31^ Our findings in Peru demonstrated that although Spo_2_ was significantly lower in children with pneumonia when compared with those who did not have pneumonia, it did not add value to other diagnostic tools to identify radiographically confirmed clinical pneumonia. One possibility that may explain our findings is that our study was conducted in a tertiary referral hospital, where physicians were able to more easily recognize hypoxemia without having to use a pulse oximeter. The sample size may also limit our ability to provide adequate inferences because only 73 children (9%) had an Spo_2_ ≤ 92%. Our analysis suggests that Spo_2_ may aid in the diagnosis of pneumonia when assessing infants 2 to 11 months of age, possibly because clinical signs of hypoxemia are more difficult to ascertain in this age group.

Finally, consolidation on LUS, when added to a clinical model, pulse oximetry, and lung auscultation, had the strongest prediction values for radiographically confirmed clinical pneumonia. Children with interstitial opacities alone on LUS were less likely to have radiographically confirmed clinical pneumonia. LUS has recently been proposed as an alternative to CXR because of its high accuracy to diagnose pneumonia in both adults^14^ and children,^15^ with pediatric studies confirming these findings in low-resource communities.^16^ In addition, studies have shown that LUS is a safe alternative to CXR in children with suspected pneumonia.^32^, ^33^ Our data support these safety and efficacy trials in the use of LUS as a good predictor of radiographically confirmed clinical pneumonia. Moreover, LUS could be a substitute for CXR in settings that do not have the capability or resources to manage a CXR system.

Our study has several strengths. First, we obtained data on clinical signs and symptoms, auscultation, Spo_2_, and imaging in a large number of children with acute respiratory symptoms. Second, LUS was interpreted by practitioners blinded to clinical or CXR information to avoid potential biases in the interpretation of LUS images. Finally, this study included a variety of acute lower respiratory conditions that could be confused with pneumonia.

Our study also has some potential shortcomings. First, the study population was mostly derived from a tertiary referral center. Although children were recruited from outpatient clinics and not all children were referred for respiratory illness, the generalizability of our findings may be limited to children seeking care at tertiary medical centers. Second, we excluded children with chronic lung disease other than asthma and congenital cardiac diseases from the study, further limiting generalizability. Third, we only conducted longitudinal scans when performing LUS. It is possible that the addition of transverse scanning would have resulted in higher diagnostic performance for pneumonia.^34^ Finally, a gold standard for the diagnosis of pediatric pneumonia is not well defined, and we acknowledge that the interobserver variability in the interpretation of CXR, especially on absence of clinical findings, is high.

## Conclusions

Different algorithms, including use of signs and symptoms, laboratory data, and imaging, have been proposed to better diagnose pneumonia in children. Still, there is no consensus of which predictors have the highest yield for discrimination of radiographically confirmed clinical pneumonia, and results may vary depending on population, age, and setting. Our analysis found that lung auscultation and LUS may improve diagnosis of pediatric pneumonia, beyond clinical signs and symptoms. The next steps should be validation studies to assessing utility, ease of use, and feasibility of auscultation and LUS tools in resource-limited settings, and their impact on clinical outcomes.
